# Relationship between fundus sex index obtained using color fundus parameters and body height or axial length in the Kumejima population

**DOI:** 10.1007/s10384-024-01082-2

**Published:** 2024-07-31

**Authors:** Takehiro Yamashita, Ryo Asaoka, Aiko Iwase, Hiroshi Sakai, Hiroto Terasaki, Taiji Sakamoto, Makoto Araie

**Affiliations:** 1 Department of Ophthalmology, Kagoshima University Graduate School of Medical and Dental Sciences, Kagoshima, Japan; 2 Department of Ophthalmology, Seirei Hamamatsu General Hospital, Shizuoka, Japan; 3 Tajimi Iwase Eye Clinic, Gifu, Japan; 4 Urasoe Sakai Eye Clinic, Okinawa, Japan; 5 Department of Ophthalmology, Kanto Central Hospital, Tokyo, Japan

**Keywords:** Fundus sex index, Fundus photographs, Axial length

## Abstract

**Purpose:**

To investigate the relationship between the fundus sex index obtained from fundus photographs and body height or axial length in the Kumejima population.

**Study Design:**

Prospective cross-sectional observational population study.

**Methods:**

Using color fundus photographs obtained from the Kumejima population, 1,653 healthy right eyes with reliable fundus parameter measurements were included in this study. The tessellation fundus index was calculated as R/(R + G + B) using the mean value of the red-green-blue intensity in the eight locations around the optic disc and foveal region. The optic disc ovality ratio, papillomacular angle, and retinal vessel angle were quantified as previously described. The masculine or feminine fundus was quantified using machine learning (L2 regularized binominal logistic regression and leave one out cross validation), with the range of 0–1 as the predictive value, and defined as the fundus sex index. The relationship between the fundus sex index and body height or axial length was investigated using Spearman’s correlation.

**Results:**

The mean age of the 838 men and 815 women included in this study was 52.8 and 54.0 years, respectively. The correlation coefficient between fundus sex index and body height was − 0.40 (*p* < 0.001) in all, 0.01 (*p* = 0.89) in men, and − 0.04 (*p* = 0.30) in women, and that between fundus sex index and axial length was − 0.23 (*p* < 0.001) in all, − 0.12 (*p* < 0.001) in men, and − 0.13 (*p* < 0.001) in women.

**Conclusion:**

This study shows that a larger number of masculine fundi tend to have longer axial lengths in each sex group. However, sex index was not significantly related with body height either in men or in women.

**Supplementary Information:**

The online version contains supplementary material available at 10.1007/s10384-024-01082-2.

## Introduction

Artificial intelligence (AI), especially deep-learning AI, could estimate age with a mean absolute error (MAE) of 3.26 years, sex with a high area under the receiver operating characteristic curve (AUC) of 0.97, systolic blood pressure within an MAE of 11.23 mmHg, smoking habits (AUC = 0.71), and the presence of cardiac complications (AUC = 0.70) within the UK biobank and EyePACS dataset, utilizing inception-v3 model [1]. AI could also measure refraction (MAE: 0.56 diopter) using the TensorFlow framework based on data from the UK biobank and the Age-Related Eye Disease Study dataset [2]. In the context of the Beijing Eye study dataset, the Inception-Resnet-v2 model could predict axial length with an MAE of 0.56 mm [3], solely based on fundus photographs.

In particular, sex could be estimated with a probability of 97% [1]. However, identifying which factors are important in estimating individuals’ sex proved impossible, even when using attention heat maps or quantifying a few factors. Similar problems occur in the games of chess, Go, and Shogi. Deep-learning AI can predict the best move (the result); however, why the move is superior remains unknown [4]. Therefore, professional chess players need to study the reasons for AI moves. However, it is difficult for professionals to clarify the reason deep learning AI selects its moves; therefore, it is called black-box AI [5].

Conventional statistical methods, such as multiple regression analysis using many factors, have been proposed as possible methods for explaining AI making it possible to unravel this black box [5]. The ocular fundus is unique and has many different features. For example, there are large individual variations in the angle or trajectory of retinal vessels [6–10], the optic disc’s location and shape [11, 12], and the color of the peripapillary area [13–16]. Specifically, the trajectory of the arcade vessels correlates with the trajectory of infra- and supra-temporal thick retinal nerve fiber layer, and both tend to move closer to the fovea as the axial length increases [6–10]. We reported that L2 regularization ridge regression using these fundus parameters could determine sex with an accuracy rate of 77.9% in young adults (20s) [17], 63.2% in those aged 8.5 years [18], and 80.4% in those aged > 40 years [19]. Because multiple regression analysis was used, sex-specific differences were also found. The optic disc was oval-shaped, retinal vessels were closer to the fovea, and the peripapillary color of the fundus was more greenish in women compared with men [17–19].

**Fig. 1:**
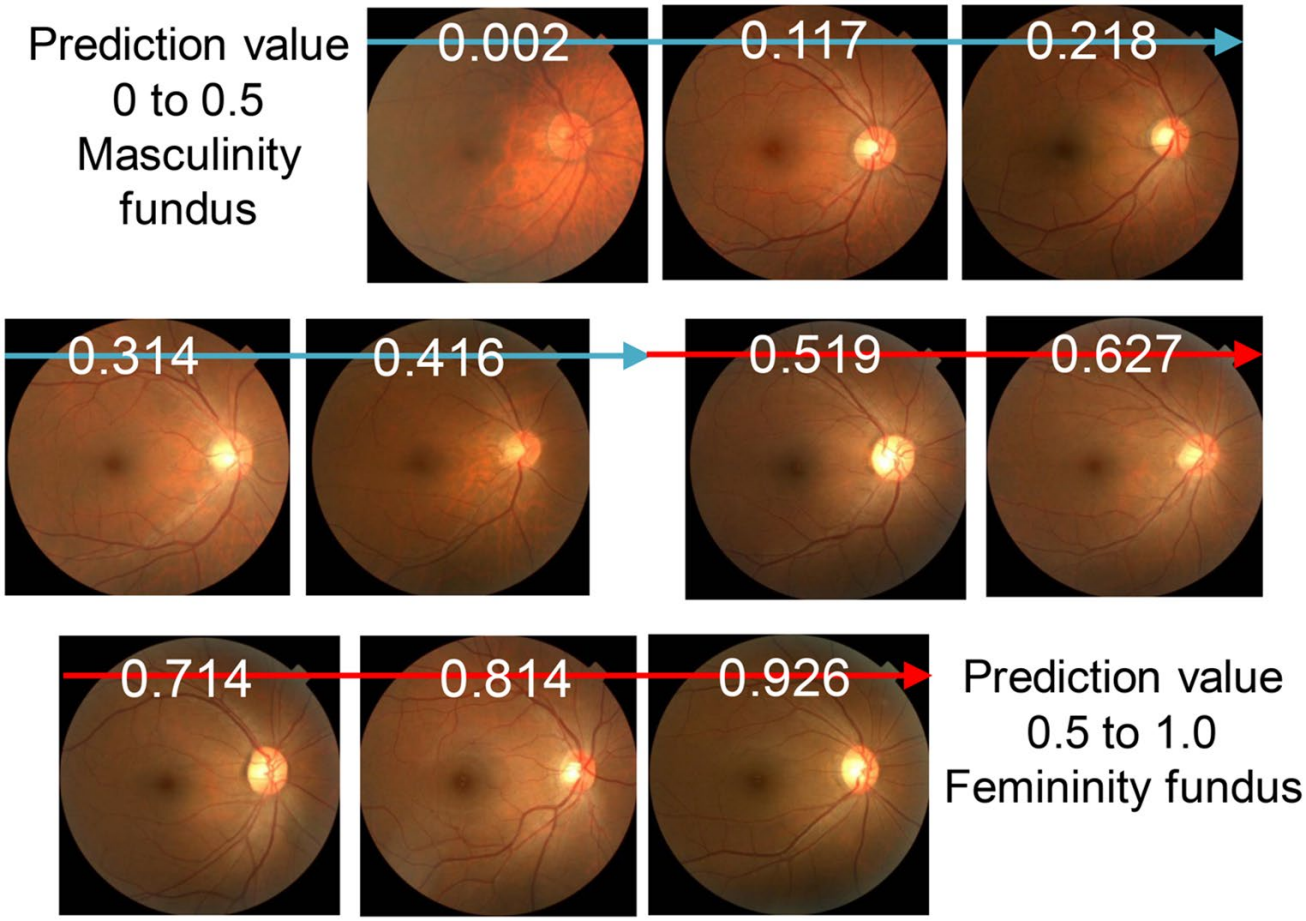
Predictive value (fundus sex index) and representative color fundus photographs of men with a masculine fundus and women with a feminine fundus. A predictive value of 0–1 calculated for each fundus photograph using multiple biomarkers with the regression analysis, with 0–0.5 indicating a masculine fundus and 0.5–1 indicating a feminine fundus

**Fig. 2:**
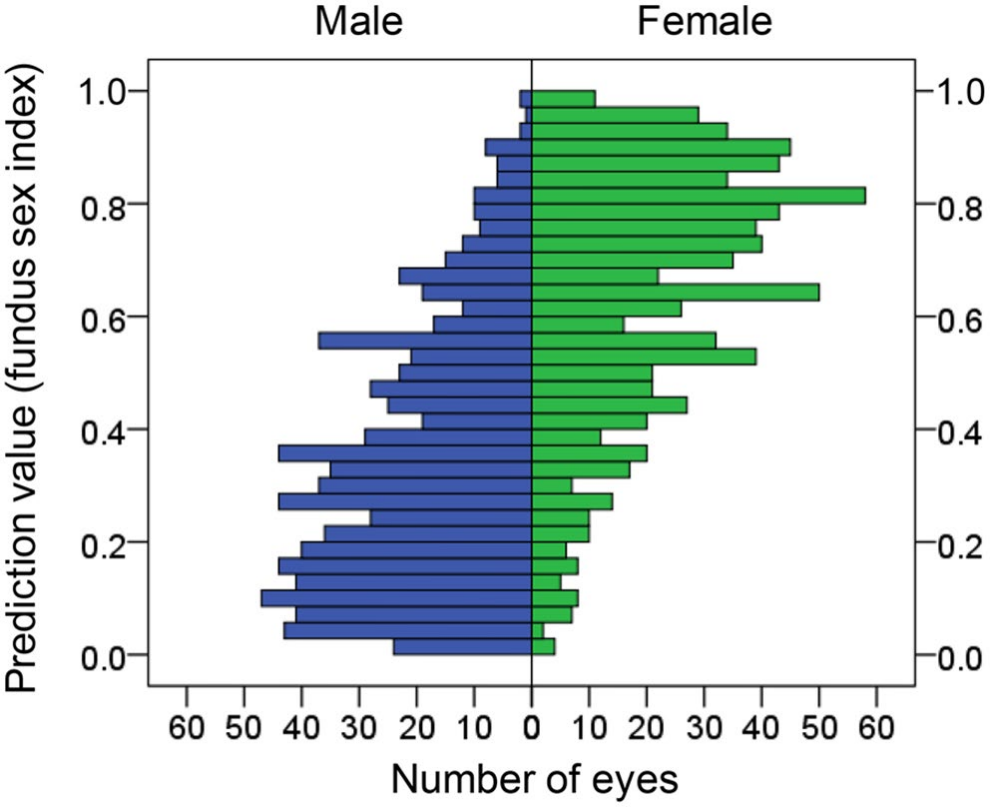
Distribution of predictive value (fundus sex index) in men and women in the Kumejima population. The fundus sex index for males tends to be low, and that for females tends to be high. Some men had a “feminine” fundus with a high predictive value, whereas some women had a “masculine” fundus with a low predictive value

A previous study shows a predictive value of 0–1 calculated for each fundus photograph using regression analysis, with 0–0.5 indicating a masculine fundus and 0.5–1 indicating a feminine fundus (Fig. 1). Figure 2 shows the distribution of predicted values obtained from the Kumejima population (The size of Kumejima island is 63.2 km2. It is located in the southwestern part of Japan, west of the main island of Okinawa.) [19]. Eyes with a predictive value close to 0 indicated a more masculine fundus, whereas those with a predictive value close to 1 indicated a more feminine fundus. This implies that sex can be quantified as a continuous variable for men or women based on fundus parameters rather than as a binary gender variable for men or women. We hypothesized that statistics-based probability (predictive value) could be used to examine sex tendencies rather than sex differences. For example, body height and axial length have significant sex-based differences, with men being taller and having longer axial lengths than women. However, some women are taller with a longer axial length, whereas some men are shorter with a shorter axial length. Using the predictive values, we examined the correlation between sex and body height or axial length in men and women separately.

In the Kumejima population, men tended to have a low predictive value (fundus sex index) and a masculine fundus, whereas women had a high predictive value and a feminine fundus. Furthermore, some men had a “feminine” fundus with a high predictive value, whereas some women had a “masculine” fundus with a low predictive value (Fig. 2). Assuming that this predictive value correlates with sex-differentiated body height and axial length in a male-only or female-only population, we considered whether this predictive value might be an indicator of masculinity or femininity.

It is unclear what the masculine and feminine values calculated from these fundus photographs represent. However, since they may represent sex tendencies, we defined the prediction value as “fundus sex index” and investigated their relationship to sex-differentiated body height and axial length in the Kumejima population.

## Methods

### Study population

The procedures conformed to the tenets of the Declaration of Helsinki and the regulations of Japan, and the protocol was approved by the Ethics Board of the Regional Council. Written informed consent was obtained from all participants before the examinations.

This study was conducted between May 2005 and August 2006, and all residents aged ≥ 40 years were informed of the protocol and invited to participate. According to the official household registration database, Kumejima had 5249 residents aged ≥ 40 years in 2005. After excluding 617 residents who could not be examined for various reasons, 4632 were eligible for the study [20, 21]. The exclusion criteria were: those with inadequate fundus photographs; eyes that had undergone pseudophakic or aphakic procedures; eyes with spherical equivalent refraction less than − 8 or exceeding + 5 diopters; subjects with optic, retinal, or brain disorders; those in whom the peripheral fundus area lacked clarity; and eyes with unmeasurable fundus parameters [19].

### Examinations and diagnosis

The screening examination consisted of a structured interview with details published elsewhere [20, 21]. Weight was determined using a digital weight scale, and height was determined using an analog stadiometer. Ocular examinations were performed by experienced ophthalmologists and examiners, and uncorrected and best-corrected visual acuity (BCVA), refractive error (spherical equivalent), intraocular pressure (IOP), central corneal thickness (CCT), anterior chamber depth (ACD), and axial length were measured. In addition, slit-lamp biomicroscopy, gonioscopy, ophthalmoscopy, fundus photography, and perimetry were performed. Sequential stereoscopic color fundus photographs (CFPs) at 30° and 45° were obtained using a non-mydriatic digital ocular fundus camera system (ImageNet TRC-NW7; Topcon). The refractive error was measured using an auto-refractometer (ARK-730, Topcon), the IOP was measured using a Goldmann applanation tonometer, and the median value was used. The CCT was measured using specular microscopy (SP-2000, Topcon), and the central ACD and axial length were measured using IOLMaster (Carl Zeiss Meditec). Peripheral ACD was scored according to the van Herick method, and gonioscopic findings were scored according to Shaffer’s grading system using a Goldmann two-mirror lens. VF was examined using the frequency doubling technology (FDT) perimetry with the C-20-1 screening program (Carl Zeiss Meditec). If ocular abnormalities were suspected, participants were referred for a more comprehensive examination. This examination included detailed slit-lamp biomicroscopy, gonioscopy, fundus examination, and VF testing using the Humphrey Field Analyzer Central 24 − 2 Swedish interactive threshold algorithm standard Program (Carl Zeiss Meditec). The details of the optic disc, fundus, and VF examinations and glaucoma diagnosis have been reported elsewhere [20, 21].

### Measurements of fundus parameters

**Fig. 3:**
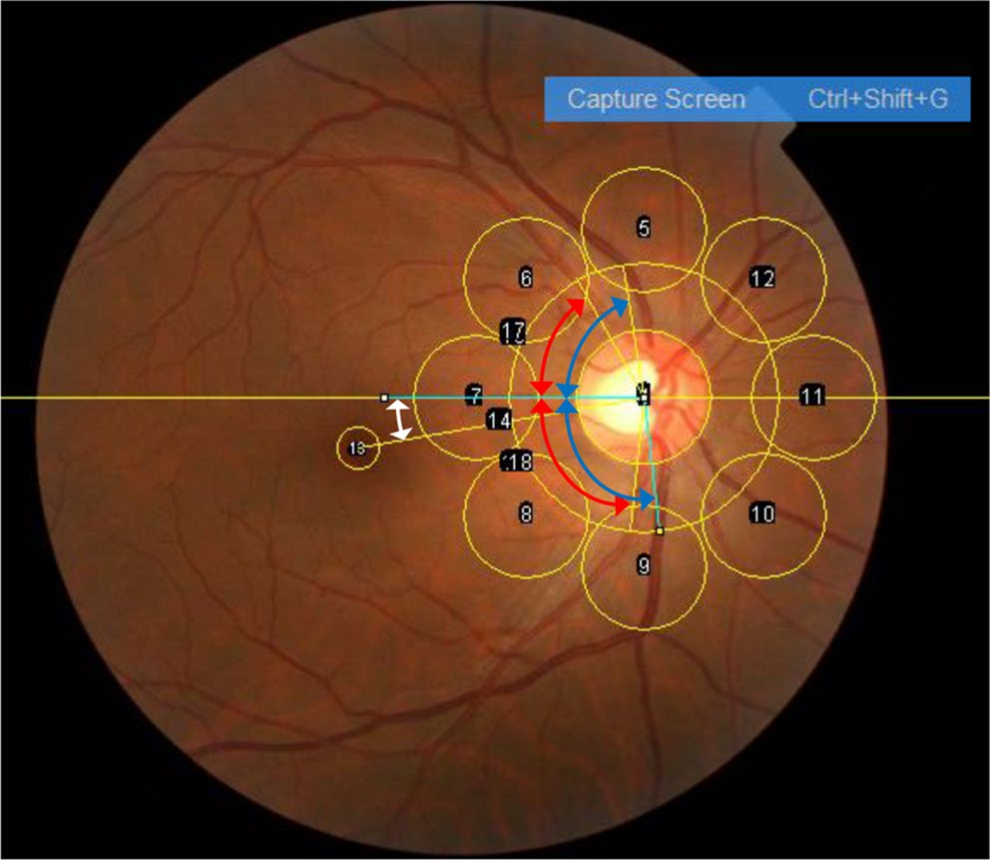
Method of quantifying retinal vessel angles and papillomacular position (PMP), ovality ratio, and red-green-blue intensity. A total of 42 fundus parameters were measured. The supratemporal (ST) or infratemporal (IT) major retinal artery angle (RA, red double arrows) or retinal vein angle (VA, blue double arrows) against the temporal horizontal line was measured (ST-RA, IT-RA, ST-VA, and IT-VA). The papillomacular position (PMP) was defined as the angle formed at the intersection of a horizontal line and a line connecting the optic disc center to the fovea (white double arrow). The ovality ratio was calculated by dividing the minimum disc diameter by the maximum. The mean values of each area’s red, green, and blue intensities were calculated for the foveal and eight peripapillary circles. The tessellation fundus index (TFI) was calculated using the mean red intensity (R), mean green intensity (G), and mean blue intensity (B) at each of the nine locations as follows: TFI = R/(R + G + B). The ovality ratio was determined by dividing the minimum optic disc diameter by the maximum. The red-green-blue intensities and the tessellation fundus index (TFI) were calculated for each of the eight locations around the optic disc and fovea. Eight 96-pixel circles were set around the optic disc, with the center of the lateral circle placed on the line between the fovea and the center of the optic nerve head. One 32-pixel circle was set on the fovea because the heat attention map of deep learning AI revealed that the foveal area was important for sex identification judgment

A total of 42 fundus parameters were measured as previously described (Fig. 3) [19]. The supratemporal (ST) or infratemporal (IT) major retinal artery angle (RA) or retinal vein angle (VA) against the temporal horizontal line was measured (ST-RA, IT-RA, ST-VA, and IT-VA) [6, 7, 10]. The papillomacular position (PMP) was defined as the angle formed at the intersection of a horizontal line and a line connecting the optic disc center to the fovea [11]. The ovality ratio was calculated by dividing the minimum disc diameter by the maximum [12]. The mean values of each area’s red, green, and blue intensities were calculated for the foveal and eight peripapillary circles. The tessellation fundus index (TFI) was calculated using the mean red intensity (R), mean green intensity (G), and mean blue intensity (B) at each of the nine locations as follows: TFI = R/(R + G + B) [13, 14].

Red double arrows indicate supratemporal and infratemporal retinal artery angles (ST-RA and IT-RA). Blue double arrows indicate supratemporal and infratemporal retinal vein angles (ST-RV and IT-RV). The white double arrow indicates PMP. The ovality ratio was determined by dividing the minimum optic disc diameter by the maximum. The red-green-blue intensities and the tessellation fundus index (TFI) were calculated for each of the eight locations around the optic disc and fovea. Eight 96-pixel circles were set around the optic disc, with the center of the lateral circle placed on the line between the fovea and the center of the optic nerve head. One 32-pixel circle was set on the fovea because the heat attention map of deep learning AI revealed that the foveal area was important for sex identification judgment [1].

These measurements were performed using CFP images and ImageJ software (ImageJ version 1.47, National Institutes of Health; available at http://imagej.nih.gov/ij/). The macro function of ImageJ enabled semi-automated calculation of the 42 CFP parameters when the locations of the fovea, edge of the optic nerve head, and crossing points between a 208-pixel circle centered on the optic disc and the superolateral and inferolateral retinal artery and vein were determined.

### Statistical analyses

As previously described, L2 regularized binomial logistic (Ridge) regression with the 42 variables, including RGB intensities and TFI in nine locations, ST-RA, IT-RA, ST-VA, IT-VA, PMP, and ovality ratio, was used to determine sex [19]. L2 regularized binomial logistic (Ridge) regression was used to overcome the overfitting problem in ordinal statistical regression models, such as multivariate or binomial logistic regression, by applying a penalty to coefficients (L2 regularization), and the sum of the absolute values of the regression coefficients was regularized [22, 23]. Specifically, the penalized version of the log-likelihood function was maximized using the following formula:

$$\sum _{i=1}^{n}\left[\right(yixi \beta -\text{log}(1+{e}_{}^{xi\beta })]- \lambda \sum _{j=1}^{p}{\beta }_{j}^{2}$$

Where xi is the i-th row of a matrix of n observations, with p predictors, β is the column vector of the regression coefficients, and λ represents the penalty applied. The diagnostic performance of the ridge binomial logistic regression approach was evaluated using the leave-one-out cross-validation method [19]. This procedure was repeated so that each eye was used once as the validation data (1,653 iterations). Diagnostic accuracy was evaluated using the area under the receiver operating characteristic curve (AROC). The final optimal model was identified using all 1,653 eyes. The masculine or feminine fundus was quantified using this formula, in the range of 0–1 as the predictive value, defined as the fundus sex index. The relationship between fundus sex index and axial length or body height was determined using Pearson’s correlation analysis. Sex differences in age, axial length, refractive error (spherical equivalent), body height, and fundus sex index were evaluated using the Mann–Whitney U test. Because the study sample was relatively large, statistical significance was set at *p* < 0.001. All statistical analyses were performed using the SPSS Statistics 19 for Windows (SPSS Inc., IBM) and R (ver. 3.1.3, The R Foundation for Statistical Computing).

## Results

Of the 4,632 eligible residents, 3,762 (81.2%) were examined. The selection was based on the right eyes primarily due to a larger number of cases where parameters could be reliably measured. Detailed inclusion and exclusion criteria and acceptable study eyes have been described previously [19, 24, 25]. Finally, 1653 right eyes (838 men and 815 women) were used for analysis. The demographic data of the right eyes of the eligible participants are shown in Table 1. The Mann-Whitney U test showed that men were significantly taller and had significantly longer axial lengths than women (*p* < 0.001). Refractive error and fundus sex index were significantly lower in men than in women (*p* < 0.001).

**Table 1:** Demographic information of the right eyes of the 1653 eligible participants

	Men	Women	*P* value
Number of eyes Age (years) Refractive error (diopters) Axial length (mm) Body height (cm) Fundus sex index	838 52.8 ± 9.4 –0.24 ± 1.47 23.71 ± 0.83 163.7 ± 6.3 0.35 ± 0.23	815 54.0 ± 10.8 –0.05 ± 1.76 23.28 ± 0.88 151.1 ± 5.8 0.64 ± 0.23	0.15
			< 0.001 < 0.001 < 0.001 < 0.001

**Fig. 4:**
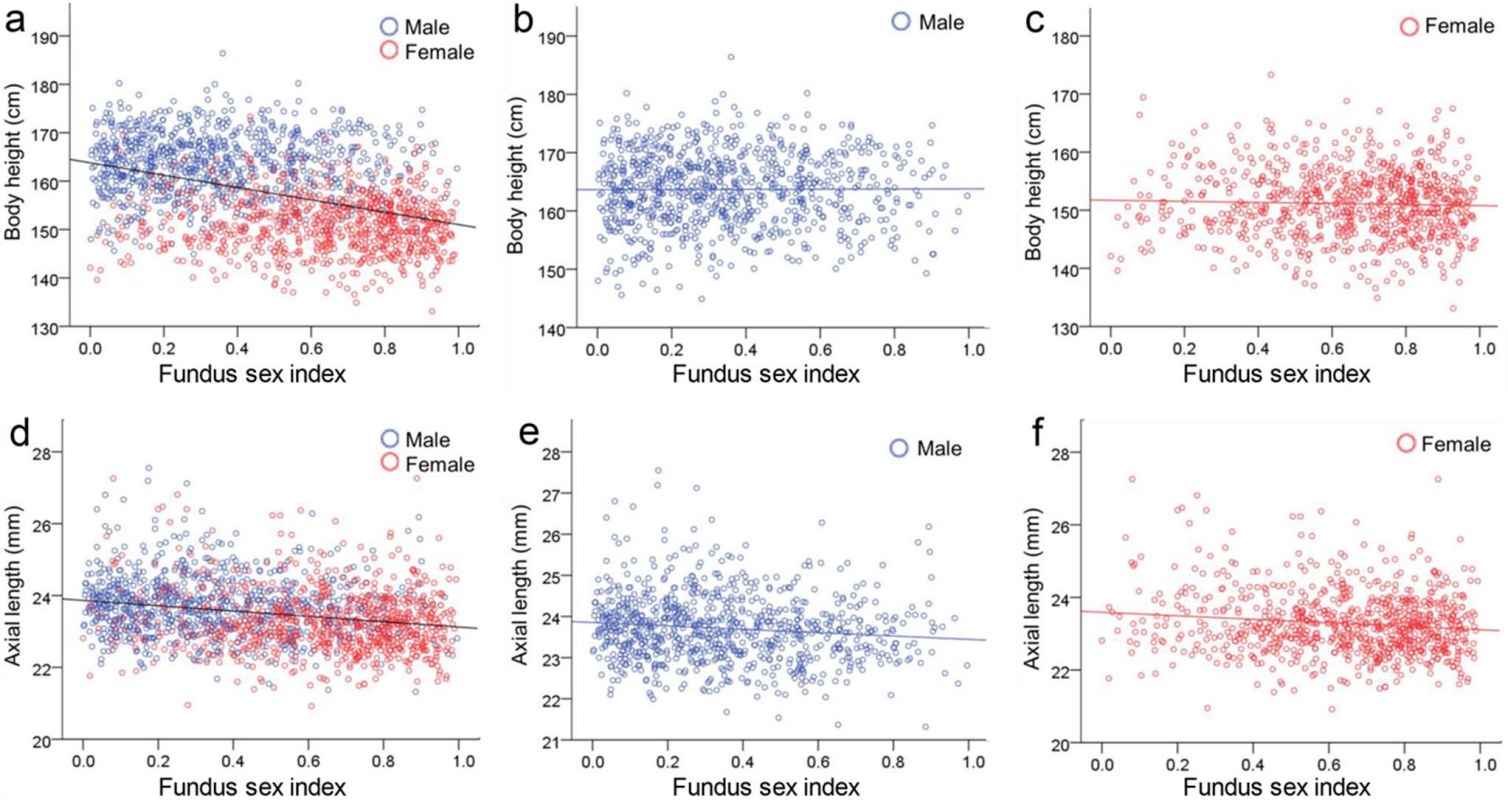
Scatter plot analysis of the relationship of fundus sex index with body height and axial length. Scatter plot analysis of the relationship between body height and fundus sex index in all individuals (*r*=–0.40, *p* < 0.001) (**a**), men (*r* = 0.01, *p* = 0.89) (**b**), and women (*r*=–0.04, *p* = 0.30) (**c**). Scatter plot analysis of the relationship between axial length and fundus sex index in all individuals (*r*=–0.23, *p* < 0.001) (**d**), men (*r*=–0.12, *p* < 0.001) (**e**), and women (*r*=–0.13, *p* < 0.001) (**f**)

The correlation coefficients between fundus sex index and body height were − 0.40 (*p* < 0.001) in all, 0.01 (*p* = 0.89) in men, and − 0.04 (*p* = 0.30) in women. The correlation coefficients between the fundus sex index and axial length were − 0.23 (*p* < 0.001) in all, − 0.12 (*p* < 0.001) in men, and − 0.13 (*p* < 0.001) in women (Fig. 4).

## Discussion

A comparison between the male and female groups indicated that men were significantly taller and had significantly longer axial lengths than women. An analysis of the fundus sex index calculated using fundus parameters showed that participants with a masculine fundus tended to be taller and had a longer axial length than those with a feminine fundus.

In the analysis of men and women separately, the eyes of participants with a more masculine fundus sex index had a longer axial length. These results were considered statistically significant. The overall correlation coefficient between fundus sex index and axial length was − 0.23, while it was − 0.12 for men only and − 0.13 for women only, indicating that the fundus sex index was affected by the axial length to a similar extent for both sexes. Because the fundus sex index is calculated using ocular fundus parameters, it may easily correlate with axial length, an eye characteristic.

Fundus parameters used to calculate the fundus sex index included the tessellation fundus index [14] and blood vessel angles [6] as factors correlated with the axial length and potential confounding factors. Stepwise multiple regression analysis for axial length can be used to investigate the confounding factors. Using fundus sex index and all fundus parameters, our results show that, when including all participants, sex index was correlated with axial length independent of other variables (standardized coefficient: -0.252, *p* < 0.001, supplementary Table 1) and in women (standardized coefficient: -0.175, *p* < 0.001, supplementary Table 3). However, the fundus sex index was not selected as an independent variable in men (supplementary Table 2). Based on this study alone it is, therefore, unsafe to conclude that there exists a relationship between fundus sex index and axial length in men. In the future, it will be necessary to conduct confirmatory research using other factors such as facial sex index or fundus sex index using AI, which can determine sex more accurately.

The overall correlation coefficient between body height and fundus sex index was − 0.40, 0.01 for men and − 0.04 for women. Because body height significantly correlates with axial length [24] and varies significantly between men and women, we expected to find separate significant correlations for men and women. However, the results show that the correlation coefficient between the fundus sex index and height was approximately zero. This indicates large physical differences between men and women; however, they do not significantly affect the sex tendency in color fundus photographs. Regression analysis with fundus sex index as the dependent variable and height and sex as explanatory variables revealed standardized coefficients of 0.01 for height (*p* = 0.56) and 0.29 for sex (*p* < 0.001), indicating that there is no relationship between the fundus sex index and body height, even though there are sex differences. It is plausible that a different sex index calculated based on physical characteristics, such as facial features [26], may correlate with body height.

The lack of correlation between fundus sex index and body height can be partly attributed to the distinct manner in which sex differences manifest during the growth phase. Body height in boys and girls was similar at ages 6.5–9.5 years [27]. The most significant factor causing body height differences between men and women is the growth spurt during the manifestation of secondary sexual characteristics [28]. On the other hand, axial length differences between boys and girls at age 8.5 are substantial [29], with boys consistently exhibiting longer axial lengths until the age of 18, without the rapid elongation observed in the growth spurt of body height [30]. Additionally, at age 8.5, gender-based distinctions in fundus parameters are noted, such as the ovality ratio being smaller in girls and higher green and blue intensities in the nasal area of the optic disc compared to boys [18]. In adults in their 20s, these differences persist, with women displaying greater green and blue intensities superiorly and temporally in the optic disc; also, the supratemporal artery was closer to the fovea compared to men. The male group showed a stronger tessellation around the optic disc than the female group [17]. These fundus variations during growth are primarily associated with axial elongation [9]. Therefore, the gender differences in body height caused by the growth spurt during secondary sexual characteristics may not be related to the fundus sex index calculated using fundus parameters that occur before and after these characteristics without the accompanying growth spurt effect.

The attention map of deep learning AI reveals that the foveal area is important for sex identification judgment [1]. Previous studies demonstrate that foveal retinal thickness and subfoveal choroidal thickness are significantly smaller in women than in men [31, 32]. Although OCT measurements were not performed in the Kumejima study, foveal green intensity was lesser in men than in women, but the distribution of foveal green intensity between the two groups overlapped [19]. Additionally, foveal TFI and red and blue intensities showed no significant differences between men and women. The AI attention map focused not only on the fovea but also on the entire fundus. These results suggest that a comprehensive judgment using several parameters is important for such analyses. Indicators such as the fundus sex index, facial sex index, and propensity score [33], calculated from multiple values, may be useful in future studies.

This study had some limitations. First is the accuracy of the fundus sex index. Sex was determined in 97% of the fundus photographs using AI [1], whereas multiple regression analysis determined sex in 80.4% [19], suggesting that the parameters for distinguishing sex differences in the fundus are insufficient. Deep learning AI cannot determine specific fundus sex differences, but can calculate a more accurate fundus sex index. Therefore, future research should be conducted, in order to calculate a fundus sex index with high accuracy using AI and correlate it with axial length, body height, or various individual differences. Second, this study was an epidemiological study of individuals aged ≥ 40 years and thus did not include young adults aged 20–40; therefore, the results for young adults are unknown. Third, because this study had only a Japanese population, the results may not be generalizable to other races.

In conclusion, we quantified masculine and feminine fundus as sex tendencies using fundus photographs. In the Kumejima population, more masculine fundi tended to have longer axial lengths in each sex group. The fundus sex index, which can quantify sexual tendencies in men and women, will be useful for future research on sex tendencies for various diseases and behaviors.

## Electronic supplementary material

Below is the link to the electronic supplementary material.

Supplementary Material 1

Supplementary Material 2

Supplementary Material 3
