# Supplementary material for: Relationship between fundus sex index obtained using color fundus parameters and body height or axial length in the Kumejima population
Source: Jpn J Ophthalmol. 2024 Jul 31;68(5):586–93. doi: 10.1007/s10384-024-01082-2 (PMC11420305; doi:10.1007/s10384-024-01082-2)
Supplement: Supplementary file 1 — Supplementary Material 1 [file 10384_2024_1082_MOESM1_ESM.docx]

**Relationship between fundus sex index obtained using color fundus parameters and body height or axial length in the Kumejima population**

Supplemental Table1. Stepwise multiple regression analysis for axial length in all participants

|  | Standardized coefficient | *P* value |
| --- | --- | --- |
| Superior green intensity | -0.264 | <0.001 |
| **Fundus sex index** | **-0.252** | **<0.001** |
| Temporal red intensity | 0.250 | <0.001 |
| Infra nasal green intensity | -0.468 | <0.001 |
| Infra temporal retinal vein angle | -0.110 | <0.001 |
| Supra temporal retinal artery angle | -0.087 | <0.001 |
| Infra temporal retinal vein angle | -0.062 | 0.006 |
| Nasal green intensity | 0.743 | <0.001 |
| Infra nasal tessellation fundus index | -0.211 | <0.001 |
| Temporal green intensity | 0.239 | 0.001 |
| Inferior blue intensity | -0.160 | <0.001 |
| Supra nasal green intensity | -0.319 | <0.001 |
| Foveal tessellation fundus index | 0.073 | 0.008 |
