# Supplementary material for: Relationship between fundus sex index obtained using color fundus parameters and body height or axial length in the Kumejima population
Source: Jpn J Ophthalmol. 2024 Jul 31;68(5):586–93. doi: 10.1007/s10384-024-01082-2 (PMC11420305; doi:10.1007/s10384-024-01082-2)
Supplement: Supplementary file 2 — Supplementary Material 2 [file 10384_2024_1082_MOESM2_ESM.docx]

**Relationship between fundus sex index obtained using color fundus parameters and body height or axial length in the Kumejima population**

Supplemental Table2. Stepwise multiple regression analysis for axial length in men

|  | Standardized coefficient | *P* value |
| --- | --- | --- |
| Nasal blue intensity | 0.756 | <0.001 |
| Temporal red intensity | 0.294 | <0.001 |
| Infra nasal green intensity | -0.337 | <0.001 |
| Supra nasal blue intensity | -0.365 | <0.001 |
| Infra temporal retinal vein angle | -0.113 | <0.001 |
| Inferior green intensity | -0.360 | <0.001 |
| Infra temporal red intensity | 0.329 | 0.006 |
| Superior red intensity | -0.235 | <0.001 |
| Ovality ratio | -0.065 | 0.034 |
