# Supplementary material for: Relationship between fundus sex index obtained using color fundus parameters and body height or axial length in the Kumejima population
Source: Jpn J Ophthalmol. 2024 Jul 31;68(5):586–93. doi: 10.1007/s10384-024-01082-2 (PMC11420305; doi:10.1007/s10384-024-01082-2)
Supplement: Supplementary file 3 — Supplementary Material 3 [file 10384_2024_1082_MOESM3_ESM.docx]

**Relationship between fundus sex index obtained using color fundus parameters and body height or axial length in the Kumejima population**

Supplemental Table3. Stepwise multiple regression analysis for axial length in women

|  | Standardized coefficient | *P* value |
| --- | --- | --- |
| Foveal tessellation fundus index | 0.119 | <0.001 |
| Nasal blue intensity | 0.363 | 0.002 |
| Temporal red intensity | 0.487 | <0.001 |
| Infra temporal retinal vein angle | -0.138 | <0.001 |
| Infra nasal blue intensity | -0.757 | <0.001 |
| Temporal tessellation fundus index | -0.181 | 0.001 |
| Superior green intensity | -0.417 | <0.001 |
| Infra nasal tessellation fundus index | -0.453 | <0.001 |
| Nasal red intensity | 0.285 | 0.003 |
| **Fundus sex index** | **-0.175** | **<0.001** |
| Supra temporal retinal artery angle | -0.100 | 0.003 |
| Infra temporal retinal artery angle | -0.068 | 0.032 |
